# Functional status modifies the impact of tumor necrosis factor-alpha on depression treatment response

**DOI:** 10.1192/bjo.2025.10837

**Published:** 2025-09-16

**Authors:** Jae-Min Kim, Hee-Ju Kang, Ju-Wan Kim, Ha-Yeon Kim, Min Jhon, Ju-Yeon Lee, Sung-Wan Kim, Il-Seon Shin

**Affiliations:** Department of Psychiatry, Chonnam National University Medical School, Gwangju, Republic of Korea

**Keywords:** Depression, tumor necrosis factor-alpha, functioning, antidepressant, remission

## Abstract

**Background:**

The association between serum tumor necrosis factor-alpha (sTNF-α) levels and antidepressant treatment responses remains controversial.

**Aims:**

This study aimed to examine the impact of sTNF-α levels on 12-week antidepressant treatment outcomes, and to explore the moderating effects of functional status on this relationship in patients with depressive disorders.

**Method:**

We measured baseline sTNF-α and evaluated functional status with the Social and Occupational Functioning Assessment Scale (SOFAS) in 1086 patients undergoing stepwise antidepressant treatment. Remission, defined as a score of ≤7 on the Hamilton Rating Scale for Depression, was assessed at 12 weeks. Logistic regression analyses were performed to adjust for relevant covariates.

**Results:**

Higher sTNF-α levels were significantly associated with non-remission at 12 weeks. This association was particularly evident among patients with higher SOFAS scores, whereas no significant association was observed in patients with lower SOFAS scores. The interaction between sTNF-α levels and SOFAS scores remained significant even after adjusting for relevant covariates.

**Conclusions:**

Baseline sTNF-α levels may serve as a useful predictor of 12-week antidepressant treatment outcomes. Incorporating functional status into the predictive model enhances the accuracy of treatment response predictions.

Depressive disorders represent a significant global health burden, characterised by high prevalence and an often inadequate response to treatment despite the availability of various therapeutic interventions.^1^ Developing biomarkers that can predict antidepressant treatment response has the potential to alleviate the burden of depression by guiding more personalised treatment strategies.^2^

In recent years, the role of neuroinflammation in the pathophysiology of depression has received considerable attention.^3^ Accumulating evidence suggests that inflammatory processes may contribute significantly to depressive symptoms through various mechanisms, including disruptions in neurotransmitter systems, impairment of neurogenesis and altered neural plasticity.^4^ Tumor necrosis factor-alpha (TNF-α), a pro-inflammatory cytokine, has emerged as an important biomarker of particular interest because of its prominent role in initiating and maintaining inflammatory responses in the central nervous system.^5^ Elevated TNF-α levels have been implicated in the pathogenesis of depression, potentially influencing mood regulation and responsiveness to treatment through mechanisms such as reducing serotonin availability or inhibiting brain-derived neurotrophic factor signalling.^6^ Given these biological links, baseline TNF-α levels have been investigated as potential predictors of antidepressant treatment outcomes.^7^ However, the existing literature presents inconsistent findings; some studies have reported that elevated TNF-α levels are associated with poorer treatment outcomes,^8^ whereas others have observed no such significant relationship.^9^ A meta-analysis concluded that there is no clear evidence linking baseline TNF-α levels to antidepressant treatment response.^10^ This inconsistency highlights the need for further investigation into the potential moderators that could clarify the role of TNF-α in treatment outcomes.

One such potential moderator is the patient’s level of social and occupational functioning. Functional status has been suggested to buffer the negative impact of various biomarkers, including inflammatory markers, on mental health outcomes. Higher functioning levels may reduce the detrimental effects of inflammation on depression, possibly by supporting better adaptive coping mechanisms and resilience.^11^ Moreover, regular exercise, which is associated with higher functioning levels, has been shown to reduce baseline TNF-α levels, contributing to its overall anti-inflammatory effects.^12^ The relationship between functioning levels and inflammatory markers, such as TNF-α, in the context of depression treatment has not been widely explored, but may provide valuable insights.

Given the established links between social and occupational functioning, inflammatory cytokines and antidepressant treatment response, we hypothesise that functioning levels could modify the association between pro-inflammatory cytokines like TNF-α and treatment outcomes. However, this potential moderating effect has not yet been adequately studied. Using data from a prospective cohort of Korean patients with depressive disorders undergoing stepwise antidepressant treatment, we aimed to examine the effects of baseline serum TNF-α (sTNF-α) levels on 12-week antidepressant treatment outcomes. Specifically, we investigated whether patients’ social and occupational functioning levels modified the relationship between sTNF-α and treatment response.

## Method

### Study outline

This investigation is part of the ongoing MAKE Biomarker discovery for Enhancing antidepressant Treatment Effect and Response (MAKE BETTER) initiative. The framework for this study has previously been outlined in a protocol article,^13^ and registered with cris.nih.go.kr (identifier: KCT0001332). Participants were recruited regardless of the specific type of depression or any physical health conditions to reflect diverse clinical settings. The approach to treatment was naturalistic, allowing for the selection of antidepressants and other therapeutic measures according to patient preferences and clinician judgement, guided by established assessment intervals and criteria. Data collection included sociodemographic and clinical characteristics at the outset, as well as treatment-related information during follow-up, captured via a structured clinical report form. Data recording and collection were conducted by clinical research coordinators who were not informed about the treatment approaches and were trained by the study’s psychiatric researchers in accurate data handling. The study was conducted in accordance with the Helsinki Declaration of 1975, as revised in 2013. Ethical approval for the study was granted by the Chonnam National University Hospital Institutional Review Board (CNUH) (approval number 2012-014). All patients gave written informed consent to participate in the study and use their data.

### Participants

Between March 2012 and April 2017, individuals were recruited from the out-patient psychiatric service at CNUH to participate in a study on the efficacy of new antidepressant therapies for both initial and repeated episodes of depression. The recruitment aimed to gather a cohort suitable for exploring biomarkers that could predict responses to antidepressant treatments, and all participants provided informed consent to undergo treatment exclusively with antidepressant medications. The study included adult participants aged 18 years or older, who met the criteria for major depressive disorder, dysthymic disorder or depressive disorder not otherwise specified, as defined by the DSM-IV-TR.^14^ Eligibility required a score of at least 14 on the Hamilton Rating Scale for Depression (HRSD).^15^ Exclusion criteria were stringent, excluding those with severe comorbid medical or psychiatric conditions, a history of organic psychosis or pregnancy. All enrolled participants, and guardians in the case of minors, signed written informed consent.

### Exposure variables

#### sTNF-α level

Participants were required to abstain from food (water was permitted) overnight before the collection of blood samples. To ensure relaxation and stabilise physiological parameters, participants were also asked to rest quietly for 25–45 min before venipuncture. sTNF-α concentrations were quantified with the Human TNF-α Quantikine HS ELISA Kit (HSTA00D, R&D Systems, Minnesota, USA) at the Global Clinical Central Laboratory in Yongin, Korea. For analytical purposes, sTNF-α levels were categorised into low and high groups based on the cohort’s median value, and were additionally examined as a continuous measure in subsequent analyses.

#### Functioning level evaluation

The assessment of social and occupational functioning was conducted with the Social and Occupational Functioning Assessment Scale (SOFAS), as defined in the DSM-IV-TR.^14^ The SOFAS score, which is designed to measure functioning independently of the individual’s psychological or physical health issues, ranges from 1 to 100, where higher scores denote better functioning. These scores were subsequently grouped into higher (>60) and lower (≤60) functioning categories, based on established SOFAS criteria that associate a score of 60 with moderate symptoms and noticeable difficulties in functioning. Additionally, the scores were treated as a continuous variable in further analyses.

### Baseline covariates

Sociodemographic data collected at baseline included age, gender, years of formal education, marital status (married or unmarried), cohabitation status (living alone or with others), religious affiliation (observing or not observing), current employment status and monthly income (above or below US$2000). Clinical characteristics assessed encompassed diagnoses of depressive disorders as previously described, with additional details such as specifiers, age at initial onset, overall duration of illness, number of depressive episodes, duration of the current episode, family history of depression and the number of comorbid physical conditions assessed via a comprehensive health questionnaire covering 15 different systems. Additionally, body mass index (BMI) and smoking status were recorded. Symptomatic assessment was conducted with the Hospital Anxiety and Depression Scale depression and anxiety subscales (HADS-D and HADS-A),^16^ and the Alcohol Use Disorders Identification Test (AUDIT),^17^ with higher scores reflecting more severe symptoms.

### Stepwise psychopharmacotherapy

The comprehensive treatment protocol employed in this study, including stepwise strategies, has been detailed in earlier publications.^18^ In brief, the treatment began with an initial antidepressant monotherapy. Adjustments were made at 3-week intervals, continuing up to 12 weeks, based on the evaluation of treatment responses and the occurrence of side-effects.

### Outcome

Remission was determined by a HRSD score of 7 or less, assessed during sequential evaluations at 3, 6, 9 and 12 weeks. For inclusion in the final analysis, participants were classified as ’remitted’ if they achieved remission at the 12-week assessment, regardless of their remission status at earlier evaluations at 3, 6 or 9 weeks. Participants who did not meet the remission criterion at the 12-week assessment were categorised as ‘non-remitted,’ even if they had achieved remission at any previous checkpoint.

### Statistical analysis

The baseline characteristics of patients were analysed based on their sTNF-α levels divided at the median (lower versus higher), SOFAS scores (>60 *v*. ≤60) and remission status (remission versus non-remission), using independent *t*-tests for continuous variables and chi-squared tests for categorical variables. The selection of covariates for adjusted analyses was informed by their statistical significance (*P* < 0.05) and the assessment of collinearity. Correlations between sTNF-α and SOFAS scores were determined with the Spearman’s rank-order correlation method. The impact of sTNF-α levels on the likelihood of 12-week remission was evaluated through logistic regression analyses, both unadjusted and adjusted for relevant covariates. Additionally, the potential interactive modifying effects of SOFAS on the relationship between sTNF-α levels and remission outcomes were explored using multinomial logistic regression with similar adjustments. Since gender differences in immune responses have been reported,^19^ we additionally explored potential differences in the associations and interactions by gender. All statistical tests were conducted as two-sided, with a significance threshold set at *P* < 0.05. The analyses were performed with IBM SPSS Statistics, version 27 for Windows (IBM Corp., Armonk, New York, USA; https://www.ibm.com/products/spss-statistics).

## Results

### Recruitment

Out of 1262 patients initially assessed, sTNF-α levels were successfully measured in 1094 individuals (86.7%). Among these, 1086 patients (86.1%) were followed up at least once during the 12-week treatment period. Attrition was minimal, with reasons for drop-out being a lack of treatment effect (*n* = 4) and loss to follow-up (*n* = 4). Comparative analysis of baseline characteristics revealed no significant differences between the 1086 participants who were followed and the 176 who were not included in the follow-up. At the end of the 12 weeks, 490 participants (45.1%) achieved a HRSD score of 7 or lower, indicating remission.

### Baseline characteristics by exposure and outcome

Baseline characteristics stratified by the sTNF-α median level (0.593 pg/mL) among patients who underwent up to 12 weeks of treatment are detailed in Supplementary Table 1 available at https://doi.org/10.1192/bjo.2025.10837. A higher sTNF-α level was notably associated with older age, male gender, lower education level, reduced monthly income, an older age at onset, a prolonged duration of the current episode, an increased number of physical disorders and a higher BMI. These variables were further analysed according to SOFAS scores and remission status, as presented in Table 1 and Supplementary Table 2, respectively. Lower SOFAS scores were significantly correlated with reduced religious observance, unemployment, major depressive disorder, a younger age at onset and elevated scores on the HADS-D and HADS-A. Non-remission status was significantly linked to younger age, lower monthly income, a younger age at onset, a longer duration of the current episode, non-smoking status and higher scores on the HADS-D and HADS-A. Given these findings, eight variables – age, gender, monthly income, duration of the present episode, number of physical disorders, BMI, current smoking status and HADS-A scores – were selected for inclusion in the adjusted analyses because of their statistical significance (*P* < 0.05) and to control for potential multicollinearity. Interestingly, baseline sTNF-α levels did not show a significant correlation with SOFAS scores (rho = −0.048; *P* = 0.110).

**Table 1:** Baseline characteristics according to scores on Social and Occupational Functioning Assessment Scale (SOFAS) in patients with depressive disorders (*N* = 1086)

	SOFAS >60 (*n* = 356)	SOFAS ≤60 (*n* = 890)	Statistical coefficients^a^	*P*-value
Sociodemographic characteristics				
Age, mean (s.d.), years	58.1 (14.7)	56.2 (15.3)	*t* = 1.925	0.054
Gender, *n* (%), female	249 (69.9)	613 (68.9)	χ^2^ = 0.136	0.712
Education, mean (s.d.), years	9.1 (5.0)	9.2 (4.7)	*t* = −0.232	0.817
Marital status, *n* (%), unmarried	101 (28.4)	282 (31.7)	χ^2^ = 1.312	0.252
Living alone, *n* (%)	46 (12.9)	141 (15.8)	χ^2^ = 1.701	0.192
Religious observance, *n* (%)	220 (61.8)	481 (54.0)	χ^2^ = 6.211	0.013*
Unemployed status, *n* (%)	81 (22.8)	283 (31.8)	χ^2^ = 10.060	0.002*
Monthly income, *n* (%), <US$2000	210 (59.0)	530 (59.6)	χ^2^ = 0.033	0.855
Clinical characteristics				
Major depressive disorder, *n* (%)	274 (77.0)	789 (88.7)	χ^2^ = 27.712	<0.001*
Melancholic feature, *n* (%)	44 (12.4)	142 (16.0)	χ^2^ = 2.589	0.108
Atypical feature, *n* (%)	27 (7.6)	55 (6.2)	χ^2^ = 0.816	0.366
Age at onset, mean (s.d.), years	52.8 (16.2)	50.2 (16.9)	*t* = 2.496	0.013*
Duration of illness, mean (s.d.), years	5.6 (8.5)	6.4 (9.4)	*t* = −1.279	0.201
Number of depressive episodes, mean (s.d.)	2.0 (4.7)	2.4 (5.3)	*t* = −1.368	0.171
Duration of present episode, mean (s.d.), months	7.5 (10.1)	7.3 (10.6)	*t* = 0.307	0.759
Family history of depression, *n* (%)	48 (13.5)	135 (15.2)	χ^2^ = 0.576	0.448
Number of physical disorders, mean (s.d.)	1.5 (0.5)	1.5 (0.5)	*t* = 0.612	0.400
Body mass index, mean (s.d.), kg/m^2^	23.0 (3.1)	23.2 (3.2)	*t* = −1.051	0.293
Current smoking, *n* (%)	33 (9.3)	112 (12.6)	χ^2^ = 2.717	0.099
Assessment scales, mean (s.d.) scores				
Hospital Anxiety and Depression Scale, depression subscale	11.7 (4.1)	14.4 (3.6)	*t* = −10.845	<0.001*
Hospital Anxiety and Depression Scale, anxiety subscale	10.1 (3.9)	12.4 (4.0)	*t* = −9.165	<0.001*
Alcohol Use Disorders Identification Test	5.3 (8.3)	5.4 (9.1)	*t* = −0.160	0.873

a Independent two-sample *t*-test or *χ* ^2^-test, as appropriate.

*P<0.05.

### Individual associations of the sTNF-α level and SOFAS scores on remission

The impact of baseline sTNF-α levels and SOFAS scores on remission status at 12 weeks is presented in Table 2. Elevated sTNF-α levels, analysed as both a binary and continuous variable, were significantly linked to non-remission at 12 weeks in both unadjusted and adjusted models. Similarly, lower SOFAS scores were significantly correlated with a lack of remission at 12 weeks, in analyses treating SOFAS as both a binary and continuous measure, before and after statistical adjustments. Associations stratified by gender are summarised in Supplementary Table 3. The association between elevated sTNF-α levels and non-remission at 12 weeks was not significant in either men or women, whereas the association between lower SOFAS scores and non-remission remained significant in both genders. No significant gender interactions were observed for either association (all *P*-values for interaction >0.45).

**Table 2:** Individual associations of serum tumor necrosis factor-alpha (sTNF-α) level and Social and Occupational Functioning Assessment Scale (SOFAS) scores as binary and continuous variables on the probability of 12-week remission

Exposure	Group	*n*	Remission	Odds ratio (95% CI)	
			*n* (%)	Unadjusted	Adjusted^a^
sTNF-α (binary)	Lower	542	261 (48.2)	Reference	Reference
	Higher	544	229 (42.1)	0.78 (0.62–0.99)*	0.77 (0.60–0.99)*
sTNF-α (increasing)	Not applicable	1086	Not applicable	0.75 (0.58–0.97)*	0.75 (0.57–0.98)*
SOFAS (binary)	>60	356	186 (52.2)	Reference	Reference
	≤60	890	354 (39.8)	0.60 (0.47–0.77)***	0.70 (0.53–0.92)*
SOFAS (decreasing)	Not applicable	1086	Not applicable	0.96 (0.95–0.98)***	0.97 (0.95–0.99)***

a Adjusted for age, gender, monthly income, duration of present episode, number of physical disorders, body mass index, current smoking and scores on the Hospital Anxiety and Depression Scale, anxiety subscale.

*P < 0.05, ***P < 0.001.

### Interactive modifying associations of the sTNF-α level and SOFAS scores on remission

**Fig. 1:**
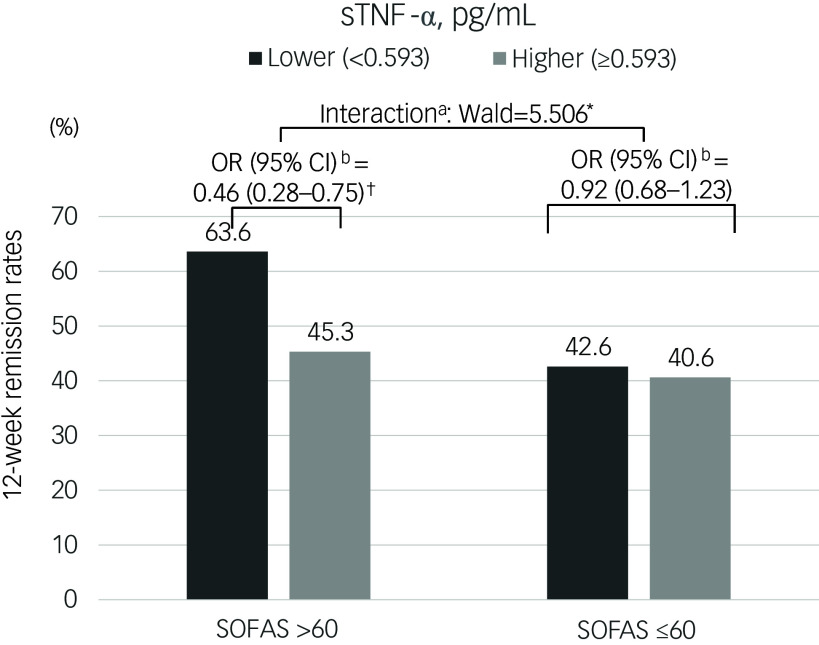
^a^Interactive effects of serum tumor necrosis factor-alpha (sTNF-α) levels and scores on remission status were estimated using multinomial logistic regression; and ^b^odds ratios (OR) (95% confidence intervals) were calculated using binary logistic regression for lower (<0.593 pg/mL) versus higher (≥0.593 pg/mL) sTNF-α levels on remission status, after adjustment for age, gender, monthly income, duration of present episode, number of physical disorders, body mass index, current smoking and scores on the Hospital Anxiety and Depression Scale-anxiety subscale. SOFAS, Social and Occupational Functioning Assessment Scale. *P < 0.05, ^†^ *P* < 0.01.

The modifying impact of SOFAS scores on the relationship between sTNF-α levels and 12-week remission outcomes is depicted in Fig. 1. In patients with higher SOFAS scores, high sTNF-α levels were significantly associated with non-remission, demonstrating a notable interaction after adjustment. Conversely, this association was not significant among patients with lower SOFAS scores, indicating a differential impact influenced by the level of social and occupational functioning. Additionally, when both sTNF-α levels and SOFAS scores were treated as continuous variables, similar interactive effects were observed, confirming the modifying role of functioning levels (Wald = 4.266; *P* = 0.045). Modifying associations stratified by gender are illustrated in Supplementary Fig. 1. Consistent with the findings in the overall sample, high sTNF-α levels were significantly associated with non-remission only among patients with higher SOFAS scores, but not among those with lower SOFAS scores, in both men and women. No significant gender interactions were observed (*P*-value for interaction = 0.810).

## Discussion

The current study, conducted within a naturalistic prospective design reflecting real-world clinical settings, observed that higher sTNF-α levels significantly affected 12-week non-remission, and this effect was notably modulated by patients’ functional levels as assessed by SOFAS scores. Despite no direct correlation between sTNF-α levels and SOFAS scores, the pronounced influence of elevated sTNF-α on treatment outcomes was evident primarily in patients with higher functional levels. This suggests that functional status may interact with biological and psychosocial factors in complex, potentially indirect ways to influence depression treatment outcomes.

Elevated proinflammatory cytokines, including TNF-α, have been linked to poorer antidepressant outcomes, potentially through mechanisms such as reduced serotonin availability or inhibition of brain-derived neurotrophic factor.^20,21^ However, findings from previous studies and meta-analyses regarding the relationship between TNF-α levels and antidepressant treatment responses have been notably inconsistent. For example, a systematic meta-analysis focusing on peripheral cytokine levels and their response to antidepressant treatment in depression revealed that only baseline IL-8 levels were consistently lower in responders compared with non-responders. In contrast, no consistent association was observed for other cytokine levels, including TNF-α.^22^ Similarly, a recent meta-analysis, which examined 11 studies involving 662 patients, found no significant differences in baseline TNF-α levels between antidepressant responders and non-responders, suggesting limited predictive value for TNF-α as a biomarker of treatment response.^23^ Further complicating this picture, some individual studies have reported divergent findings. For example, higher baseline TNF-α levels predicted a more favourable antidepressant response,^24,25^ adding further complexity to the interpretation of TNF-α as a predictive biomarker.

Our findings offer new insight into the longstanding debate by highlighting the potential moderating role of patients’ functional levels in the association between TNF-α and antidepressant response. The absence of a direct correlation between baseline TNF-α levels and SOFAS scores suggests that TNF-α may not uniformly predict clinical outcomes across all patient populations. Rather, a threshold of neuroinflammatory activity may exist, above which elevated TNF-α levels exert particularly detrimental effects, potentially in individuals with higher functional levels, as a result of their initially greater neural activity and connectivity.^4^ Higher-functioning patients might initially compensate for the neurobiological effects of inflammation, thereby masking early signs of treatment resistance.^26^ For example, individuals with higher functional levels are more likely to engage in regular physical activity, maintain stronger social networks and adhere more closely to treatment recommendations, all of which could serve as compensatory mechanisms that temporarily buffer the negative impact of inflammation.^27,28^ However, with sustained high levels of TNF-α, these compensatory mechanisms may eventually fail, leading to more pronounced negative treatment outcomes.

In contrast, the negligible impact of TNF-α on antidepressant outcomes among patients with lower functional levels could reflect several alternative possibilities. First, their substantial baseline impairment may overshadow the incremental influence of inflammation, contributing to a ‘ceiling effect’, where additional biological stressors produce minimal observable impact.^29^ Second, these individuals may exhibit reduced biological sensitivity to inflammatory changes, as a result of chronic exposure and adaptation to stress and inflammation over time.^30^ Third, lower functioning may itself represent a marker of more severe and chronic illness, associated with poorer overall treatment responsiveness,^31^ rather than reflecting a specific interaction with inflammatory processes. Furthermore, comorbid medical conditions or medications influencing inflammatory activity may have confounded these relationships,^32^ although such factors were not systematically assessed in the present study.

Additionally, the threshold effect proposed in this study could partially explain why the simple correlation (rho) between TNF-α and SOFAS was non-significant. Non-linear or more complex interaction patterns may underlie these associations, and future research utilising longitudinal biomarker measurements, non-linear modelling and detailed clinical profiling will be necessary to clarify these mechanisms.

Overall, these results underscore the complexity inherent in the interplay between inflammatory markers, psychosocial factors and antidepressant treatment response. They emphasise the necessity of examining broader functional and psychosocial dimensions alongside biological markers to refine predictive models for depression outcomes. Further research is warranted to investigate these moderating factors more comprehensively, and to inform the development of personalised treatment strategies.

Several important issues should be considered before drawing a conclusion. The measure of SOFAS used in this study may reflect multiple underlying factors beyond coping or resilience alone. For instance, functional levels assessed by SOFAS scores could also represent the chronicity and severity of depressive episodes, personality-related characteristics, psychosocial stressors or lifestyle factors such as physical activity.^27,31^ Thus, the moderating effects observed might not exclusively reflect adaptive resilience, but rather a composite effect of various psychosocial and clinical dimensions. Although we adjusted for relevant clinical and demographic variables, residual confounding by unmeasured factors cannot be completely ruled out. Further studies incorporating comprehensive measures of chronicity, personality traits, detailed psychosocial assessments and lifestyle factors will be necessary to clarify these complex relationships. Additionally, previous studies have reported that immune responses may be more pronounced in response to stress or antidepressant treatment among different genders.^19^ However, no such gender-specific differences were observed in the present study. Differences in study design, including the naturalistic approach to drug treatment and the broad age range of participants, may account for this discrepancy. Further research is needed to clarify these potential gender-related differences.

Our study’s limitations include the absence of longitudinal data on sTNF-α levels to assess associations with treatment changes and the potential variability in treatment as a result of its naturalistic design. Additionally, although we adjusted for multiple baseline clinical variables, we did not directly adjust for baseline depression severity (HRSD scores) in our main analyses. Given that remission was defined dichotomously, and that baseline severity could influence the likelihood of achieving remission, this may have introduced some bias. Future studies should further explore the independent effects of inflammatory markers and functional status, while carefully controlling for baseline depression severity. Despite these limitations, the study’s strengths lie in its large sample size, high follow-up rate and the use of standardised scales, making it a pioneering exploration of the modifying effects of functional levels on the relationship between sTNF-α and antidepressant treatment outcomes.

In conclusion, our findings highlight the multifaceted pathways through which biological, psychological and social factors intersect to affect mental health outcomes. The significant moderating effect of functional status on the sTNF-α relationship with treatment responses, even without direct correlations, illustrates the complex dynamics at play, and underscores the need for holistic approaches in depression treatment. These findings suggest that considering functional levels can enhance the predictability of sTNF-α as a biomarker for treatment outcomes. Further research is necessary to explore whether adjunctive anti-inflammatory treatments might benefit specific subpopulations identified by their TNF-α and functional profiles.

## Supporting information

Kim et al. supplementary material Kim et al. supplementary material

## Data Availability

The data that support the findings of study are available from the corresponding author (J.-M.K.), upon reasonable request.

## Supplementary material

The supplementary material is available online at https://doi.org/10.1192/bjo.2025.10837
