# Supplementary material for: Functional status modifies the impact of tumor necrosis factor-alpha on depression treatment response
Source: BJPsych Open. 2025 Sep 16;11(5):e215. doi: 10.1192/bjo.2025.10837 (PMC12451551; doi:10.1192/bjo.2025.10837)
Supplement: Kim et al. supplementary material [file S2056472425108375sup001.docx]

Functional status modifies the impact of tumor necrosis factor-alpha on depression treatment response

Jae-Min Kim^1a*^, Hee-Ju Kang^1a^, Ju-Wan Kim^1^, Ha-Yeon Kim^1^, Min Jhon^1^, Ju-Yeon Lee^1^, Sung-Wan Kim^1^, Il-Seon Shin^1^

**Supplementary Material**

Figure S1‧‧‧‧‧‧‧‧‧‧‧‧‧‧‧‧‧‧‧‧‧‧‧‧‧‧‧‧‧‧‧‧‧‧‧‧‧‧‧‧‧‧‧‧‧‧‧‧‧‧‧‧‧‧‧‧‧‧‧‧‧‧‧‧‧‧‧‧‧‧‧‧‧‧‧‧‧‧‧‧‧‧‧‧‧‧‧‧‧‧‧‧‧‧‧‧‧‧‧‧‧‧‧‧‧‧‧‧‧‧‧‧‧‧‧‧‧‧‧‧‧‧‧‧‧‧2

Table S1‧‧‧‧‧‧‧‧‧‧‧‧‧‧‧‧‧‧‧‧‧‧‧‧‧‧‧‧‧‧‧‧‧‧‧‧‧‧‧‧‧‧‧‧‧‧‧‧‧‧‧‧‧‧‧‧‧‧‧‧‧‧‧‧‧‧‧‧‧‧‧‧‧‧‧‧‧‧‧‧‧‧‧‧‧‧‧‧‧‧‧‧‧‧‧‧‧‧‧‧‧‧‧‧‧‧‧‧‧‧‧‧‧‧‧‧‧‧‧‧‧‧‧‧‧‧‧‧4

Table S2 ‧‧‧‧‧‧‧‧‧‧‧‧‧‧‧‧‧‧‧‧‧‧‧‧‧‧‧‧‧‧‧‧‧‧‧‧‧‧‧‧‧‧‧‧‧‧‧‧‧‧‧‧‧‧‧‧‧‧‧‧‧‧‧‧‧‧‧‧‧‧‧‧‧‧‧‧‧‧‧‧‧‧‧‧‧‧‧‧‧‧‧‧‧‧‧‧‧‧‧‧‧‧‧‧‧‧‧‧‧‧‧‧‧‧‧‧‧‧‧‧‧‧‧‧‧‧ 6

Table S3‧‧‧‧‧‧‧‧‧‧‧‧‧‧‧‧‧‧‧‧‧‧‧‧‧‧‧‧‧‧‧‧‧‧‧‧‧‧‧‧‧‧‧‧‧‧‧‧‧‧‧‧‧‧‧‧‧‧‧‧‧‧‧‧‧‧‧‧‧‧‧‧‧‧‧‧‧‧‧‧‧‧‧‧‧‧‧‧‧‧‧‧‧‧‧‧‧‧‧‧‧‧‧‧‧‧‧‧‧‧‧‧‧‧‧‧‧‧‧‧‧‧‧‧‧‧‧‧ 8

**Fig. S1.** Interactive modifying associations of serum tumor necrosis factor-alpha (sTNF-α) levels and Social and Occupational Functioning Assessment Scale (SOFAS) scores on the probability of 12-week remission by sex.

^a^Interactive effects of sTNF-α levels and scores on remission status were estimated using multinomial logistic regression; and ^b^odds ratios (95% confidence intervals) were calculated using binary logistic regression for lower (<0.593 pg/mL) vs. higher (≥0.593 pg/mL) sTNF-α levels on remission status, after adjustment for age, monthly income, duration of present episode, number of physical disorders, body mass index, current smoking, and scores on Hospital Anxiety & Depression Scale-anxiety subscale.

^*^P <0.05, ^†^P <0.01.


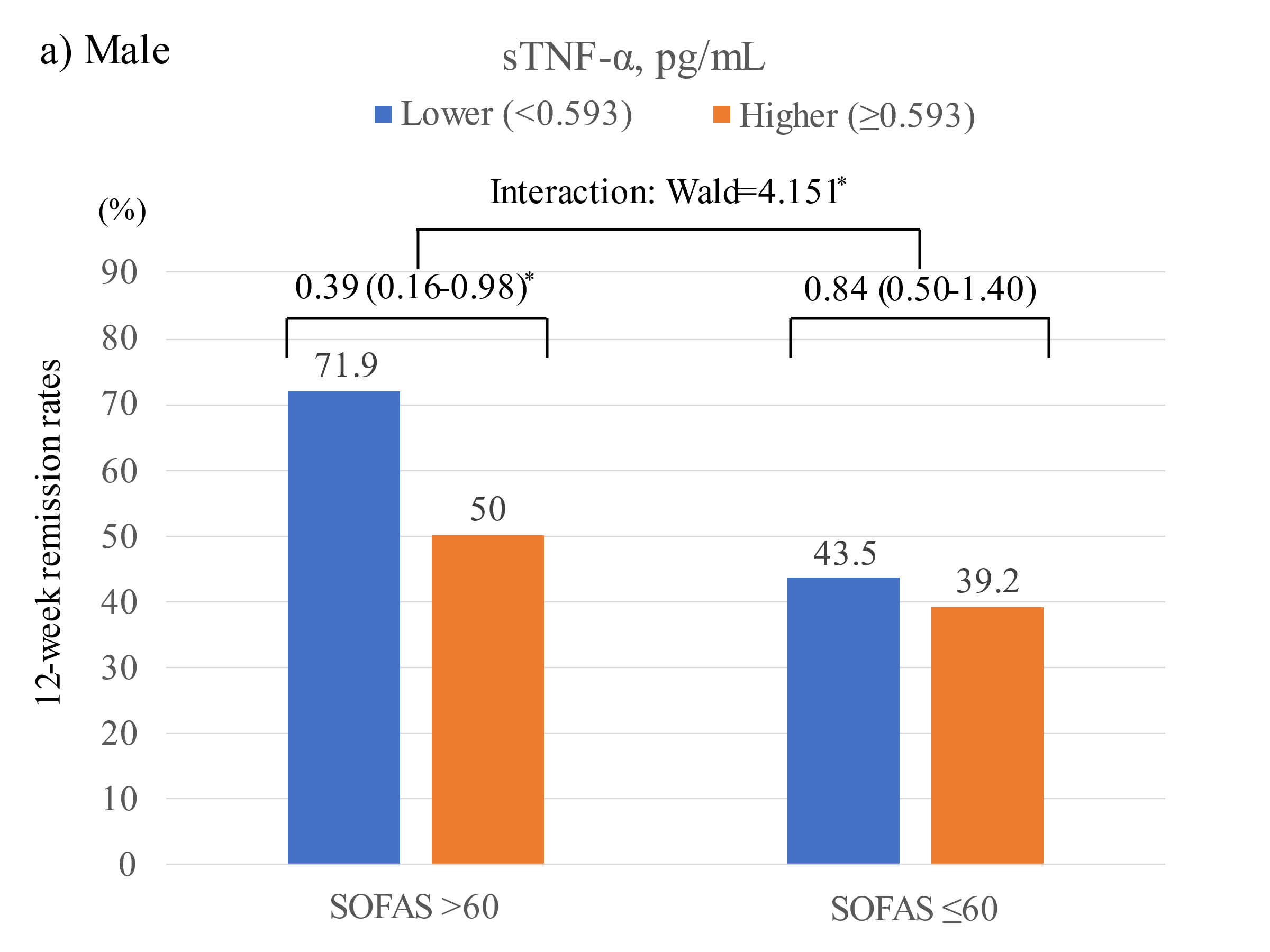


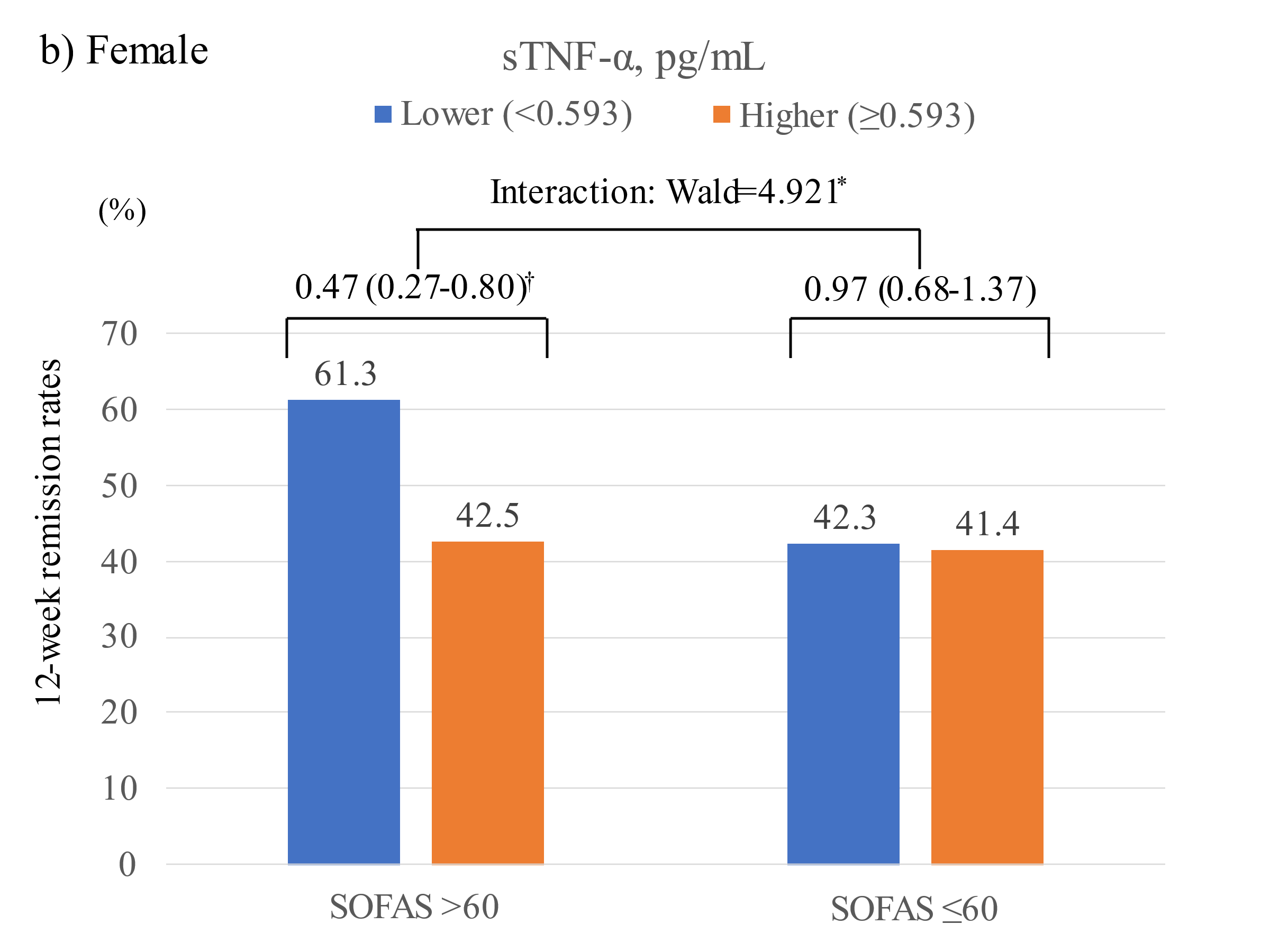


| **Table S1.** Baseline characteristics according to serum tumor necrosis factor-alpha (sTNF-α) level in patients with depressive disorders (N = 1,086). | | | | | |
| --- | --- | --- | --- | --- | --- |
|  |  | Lower sTNF-α (N = 542) | Higher sTNF-α (N = 544) | Statistical  coefficients^a^ | P-value |
| **Socio-demographic characteristics** | |  |  |  |  |
| Age, mean (SD) years |  | 54.9 (14.6) | 59.0 (14.9) | t=-4.565 | **<0.001** |
| Sex, N (%) female |  | 395 (72.9) | 350 (64.3) | χ^2^=9.192 | **0.002** |
| Education, mean (SD) years |  | 9.6 (4.5) | 8.6 (5.0) | t=+3.437 | **0.001** |
| Marital status, N (%) unmarried |  | 174 (32.1) | 152 (27.9) | χ^2^=2.239 | 0.135 |
| Living alone, N (%) |  | 89 (16.4) | 78 (14.3) | χ^2^=0.905 | 0.342 |
| Religious observance, N (%) |  | 299 (55.2) | 308 (56.6) | χ^2^=0.232 | 0.630 |
| Unemployed status, N (%) |  | 152 (28.0) | 164 (30.1) | χ^2^=0.582 | 0.446 |
| Monthly income, N (%) <2,000 USD |  | 299 (55.2) | 349 (64.2) | χ^2^=9.115 | **0.003** |
| **Clinical characteristics** |  |  |  |  |  |
| Major depressive disorder, N (%) |  | 458 (84.5) | 467 (85.8) | χ^2^=0.388 | 0.533 |
| Melancholic feature, N (%) |  | 76 (14.0) | 86 (15.8) | χ^2^=0.683 | 0.409 |
| Atypical feature, N (%) |  | 41 (7.6) | 28 (5.1) | χ^2^=2.667 | 0.102 |
| Age at onset, mean (SD) years |  | 49.3 (15.9) | 52.5 (17.0) | t=-3.109 | **0.001** |
| Duration of illness, mean (SD) years |  | 5.9 (8.2) | 6.7 (10.3) | t=-1.526 | 0.127 |
| Number of depressive episodes, mean (SD) |  | 2.3 (4.9) | 2.3 (5.2) | t=-0.220 | 0.826 |
| Duration of present episode, mean (SD) months |  | 6.5 (9.5) | 8.3 (11.2) | t=-2.786 | **0.005** |
| Family history of depression, N (%) |  | 79 (14.6) | 79 (14.5) | χ^2^=0.001 | 0.980 |
| Number of physical disorders, mean (SD) |  | 1.4 (0.5) | 1.6 (0.5) | t=-3.600 | **<0.001** |
| Body mass index, mean (SD) kg/m^2^ |  | 22.9 (3.2) | 23.5 (3.1) | t=-3.144 | **0.002** |
| Current smoking, N (%) |  | 52 (9.6) | 71 (13.1) | χ^2^=3.231 | 0.072 |
| **Assessment scales, mean (SD) scores** | |  |  |  |  |
| Hospital Anxiety & Depression Scale-depression subscale |  | 13.7 (3.9) | 13.6 (4.0) | t=+0.302 | 0.762 |
| Hospital Anxiety & Depression Scale-anxiety subscale |  | 11.9 (4.0) | 11.7 (4.1) | t=+1.100 | 0.272 |
| Alcohol Use Disorder Identification Test |  | 4.9 (8.2) | 5.8 (9.5) | t=-1.777 | 0.076 |
| ^a^Independent two-sample t-test or χ^2^ test, as appropriate.  **Bold style** denotes statistical significance (p-values<0.05). | | | | | |

| **Table S2.** Baseline characteristics by 12-week remission status in patients with depressive disorders (N = 1,086). | | | | | |
| --- | --- | --- | --- | --- | --- |
|  |  | Remission  (N = 490) | No remission (N = 596) | Statistical  coefficients^a^ | P-value |
| **Socio-demographic characteristics** | |  |  |  |  |
| Age, mean (SD) years |  | 58.2 (13.9) | 55.9 (15.6) | t=-2.610 | **0.009** |
| Sex, N (%) female |  | 334 (68.2) | 411 (69.0) | χ^2^=0.079 | 0.778 |
| Education, mean (SD) years |  | 9.1 (4.9) | 9.1 (4.7) | t=+0.065 | 0.948 |
| Marital status, N (%) unmarried |  | 134 (27.3) | 192 (32.2) | χ^2^=3.033 | 0.082 |
| Living alone, N (%) |  | 73 (14.9) | 94 (15.8) | χ^2^=0.158 | 0.691 |
| Religious observance, N (%) |  | 281 (57.3) | 326 (54.7) | χ^2^=0.765 | 0.382 |
| Unemployed status, N (%) |  | 130 (26.5) | 186 (31.2) | χ^2^=2.852 | 0.091 |
| Monthly income, N (%) <2,000 USD |  | 273 (55.7) | 375 (62.9) | χ^2^=5.801 | **0.016** |
| **Clinical characteristics** |  |  |  |  |  |
| Major depressive disorder, N (%) |  | 415 (84.7) | 510 (85.6) | χ^2^=0.164 | 0.686 |
| Melancholic feature, N (%) |  | 67 (13.7) | 95 (15.9) | χ^2^=1.088 | 0.297 |
| Atypical feature, N (%) |  | 34 (6.9) | 35 (5.9) | χ^2^=0.514 | 0.473 |
| Age at onset, mean (SD) years |  | 53.6 (15.7) | 50.5 (17.3) | t=+3.042 | **0.002** |
| Duration of illness, mean (SD) years |  | 4.7 (8.7) | 5.4 (9.3) | t=-1.311 | 0.190 |
| Number of depressive episodes, mean (SD) |  | 1.0 (1.4) | 1.2 (1.5) | t=-1.836 | 0.067 |
| Duration of present episode, mean (SD) months |  | 6.4 (8.0) | 8.3 (12.0) | t=-3.128 | **0.002** |
| Family history of depression, N (%) |  | 76 (15.5) | 82 (13.8) | χ^2^=0.664 | 0.415 |
| Number of physical disorders, mean (SD) |  | 1.7 (1.2) | 1.6 (1.3) | t=+1.212 | 0.226 |
| Body mass index, mean (SD) kg/m^2^ |  | 23.2 (3.3) | 23.2 (3.0) | t=+0.219 | 0.827 |
| Current smoking, N (%) |  | 80 (13.4) | 43 (8.8) | χ^2^=5.783 | **0.016** |
| **Assessment scales, mean (SD) scores** | |  |  |  |  |
| Hospital Anxiety & Depression Scale-depression subscale |  | 20.4 (4.1) | 21.0 (4.1) | t=-2.418 | **0.016** |
| Hospital Anxiety & Depression Scale-anxiety subscale |  | 11.3 (4.1) | 12.2 (4.0) | t=-3.666 | **< 0.001** |
| Alcohol Use Disorder Identification Test |  | 5.3 (9.2) | 5.4 (8.4) | t=-0.110 | 0.912 |
| ^a^Independent two-sample t-test or χ^2^ test, as appropriate.  **Bold style** denotes statistical significance (p-values<0.05). | | | | | |

| **Table S3.** Individual associations of serum tumor necrosis factor-alpha (sTNF-α) level and Social and Occupational Functioning Assessment Scale (SOFAS) scores as the binary variable on the probability of 12-week remission by sex. | | | | |
| --- | --- | --- | --- | --- |
| Sex | N | No. (%) | Adjusted^a^ OR (95% CI) for sTNF-α (<0.593 vs. ≥0.593 pg/mL) | Adjusted^a^ OR (95% CI) for SOFAS (>60 vs. ≤60 scores) |
| Male | 341 | 156 (45.7) | 0.76 (0.49-1.17) | 0.73 (0.56-0.94)^*^ |
| Female | 745 | 334 (44.8) | 0.79 (0.59-1.05) | 0.82 (0.71-0.96)^*^ |
| ^a^Adjusted for age, monthly income, duration of present episode, number of physical disorders, body mass index, current smoking, and scores on Hospital Anxiety & Depression Scale-anxiety subscale.  ^*^P <0.05. | | | | |
